# Odor Valence Linearly Modulates Attractiveness, but Not Age Assessment, of Invariant Facial Features in a Memory-Based Rating Task

**DOI:** 10.1371/journal.pone.0098347

**Published:** 2014-05-29

**Authors:** Janina Seubert, Kristen M. Gregory, Jessica Chamberland, Jean-Marc Dessirier, Johan N. Lundström

**Affiliations:** 1 Monell Chemical Senses Center, Philadelphia, Pennsylvania, United States of America; 2 Sensation, Perception & Behavior, Unilever R&D, Trumbull, Connecticut, United States of America; 3 Department of Clinical Neuroscience, Karolinska Institute, Stockholm, Sweden; 4 Department of Psychology, University of Pennsylvania, Philadelphia, Pennsylvania, United States of America; Technical University of Dresden Medical School, Germany

## Abstract

Scented cosmetic products are used across cultures as a way to favorably influence one's appearance. While crossmodal effects of odor valence on perceived attractiveness of facial features have been demonstrated experimentally, it is unknown whether they represent a phenomenon specific to affective processing. In this experiment, we presented odors in the context of a face battery with systematic feature manipulations during a speeded response task. Modulatory effects of linear increases of odor valence were investigated by juxtaposing subsequent memory-based ratings tasks – one predominantly affective (attractiveness) and a second, cognitive (age). The linear modulation pattern observed for attractiveness was consistent with additive effects of face and odor appraisal. Effects of odor valence on age perception were not linearly modulated and may be the result of cognitive interference. Affective and cognitive processing of faces thus appear to differ in their susceptibility to modulation by odors, likely as a result of privileged access of olfactory stimuli to affective brain networks. These results are critically discussed with respect to potential biases introduced by the preceding speeded response task.

## Introduction

Inferences of stable personal characteristics during face perception are, to a large extent, derived from so-called invariant perceptual features or semantic codes [1], [2]. These features constitute signals of high social and reproductive relevance during human interactions, and behaviors aiming to favorably influence them can be traced back to antiquity [3]. While manipulations can be achieved directly through visual emphasis or concealment of facial features, indirect manipulations through contextual cues, in particular pleasant fragrances, are very common. The cosmetic industry has long exploited the idea that certain odors enhance personal appearance, resulting in a large selection of fragranced facial and body products. In 2011 alone, the global market demand for these products was estimated at an impressive 425.8 billion US dollars [4]. Only recently, however, studies have begun to experimentally explore the mechanisms by which odors exert an influence on visual perception. In line with experimental evidence indicating that the emotional valence of olfactory cues can affect preference for previously neutral visual stimuli [5], [6], [7], [8], or emotion identification performance [9], [10], [11], studies have demonstrated that the perception of the attractiveness of facial features can be modulated by concurrent presentation of odors with either a very positive or very negative valence [12], [13]. Whether these effects are specific to affective processing, however, remains to be explored.

The present study aimed to dissociate odor-dependent modulation of emotional and cognitive aspects of invariant feature processing. Specifically, response patterns to linear modulation of odor valence and facial feature expression on two different outcome variables, age and attractiveness judgments, were explored. Both represent salient fertility cues; however, attractiveness perception has been preferentially associated with emotional processing, while age perception is thought of as a cognitive process. Attractiveness judgments form early in life, are mostly experience-independent [14], [15], and based on configural relationships between individual facial features [16]. Extraction and analysis of facial aging cues, on the other hand, constitutes an analytically driven process [17] with a relatively high cognitive load [18]. In line with these findings, face inversion tasks, which change the holistic appearance of relations between facial components while leaving individual components intact, have typically been shown to disrupt attractiveness perception, but not age processing [19], [20]. Taking advantage of the difference in perceptual processing between these tasks, we are investigating whether a similar dissociation can be observed for the influence of olfactory stimulus valence on facial feature perception.

Most studies to date have investigated multisensory effects of odors using dichotomized visual stimuli and two opposing odor valence conditions, such as one extremely pleasant and one extremely unpleasant odor. Such dichotomies fail to appropriately reflect the perceptual and emotional stimulus space we commonly experience [21], [22]. Furthermore, they do not permit the analysis of continuous transitions between extreme endpoints of stimulus space, which are highly informative about the underlying perceptual mechanisms: while additive effects between the two modalities suggest a joint representation of stimulus space, non-linear patterns and dominance of one modality over the other suggest separate representations.

Given the strong linkage between odor and affective processing, we expected to observe dissociable response patterns in age estimation and attractiveness judgments resulting from linear modulations of odor valence. More specifically, we hypothesized that judgment of attractiveness, which relies to a great extent on affective processing, would be linearly affected by the valence of a concurrently presented odor. The cognitive-analytical process of age judgment, however, would be characterized by perceptual dominance of visual feature processing and absence of additive integration.

## Materials and Methods

### Ethics Statement

**Figure 1:**
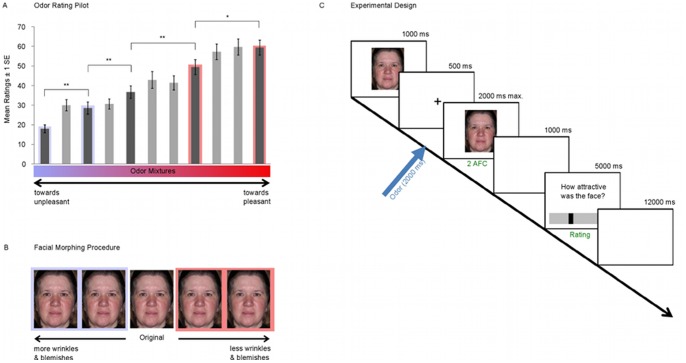
Stimulus Selection and Design. **A**. Results of odor pilot study. Valence ratings were acquired for 11 stepwise mixtures between 100% fish odor (unpleasant) and 100% Rose odor (pleasant). Five odors were chosen which perceptually corresponded to equidistant linearly increasing valence percepts (dark grey bars). All five odors were significantly different from each other (*: *p*<.05, **: *p*<.01). Red box indicates odors which were grouped together as “more pleasant” in the factorial analysis, blue box indicates odors which were grouped together as “more unpleasant”. **B**. Illustration of facial morphing procedure. The middle picture illustrates the original image; pictures framed by blue box illustrate increasing features (grouped together for factorial analysis), while pictures in the red box illustrate increasing features. **C**. Experimental Design.

All participants provided written, informed consent prior to participation, and all aspects of the study were approved by the University of Pennsylvania's Institutional Review Board (IRB) prior to starting the study and performed in accord with the Declaration of Helsinki on Biomedical Studies Involving Human Subjects. In addition, the subject depicted in figure 1 provided written informed consent, as outlined in the PLoS consent form, to publication of their photograph. To adhere to PloS standards for data availability, the dataset used to reach the conclusions drawn in the manuscript is available through the Swedish Vetenskapsrådet digital deposit center (http://snd.gu.se/sv), along with related metadata and methods, and any additional data required to replicate the reported study findings in their entirety.

### Participants

Twenty-two healthy control subjects were enrolled in the study; of these, four were excluded due to suspected malingering based on performance on the speeded response task (accuracy at chance level as defined by one-sample t-test against average performance of .5). The remaining sample consisted of 18 healthy non-smoking participants (12 women, mean age  = 25, SD = 2.7) who were instructed not to eat or drink anything but water one hour prior to testing and not to wear any scented products on the day of testing. All participants had a functional sense of smell as established by a 16-items 4 alternatives cued odor identification test [23] all >11, mean  = 13.44 *SD*±1.19).

### Odor Stimuli and Delivery

Odor stimuli with a linear progression in perceived valence from a mildly unpleasant to a mildly pleasant percept were created using an essential oil with the odor quality of fish (1% cooked fish odor; Symrise AG, Holzminden, Germany) and an essential oil smelling like roses (5% rose odor; Givaudan Inc.), both diluted in 1,2-propanediol (Sigma Aldrich, St. Louis, MO, USA). Iso-intensity of the two stimulus compounds was established in a separate pilot study on a panel of 10 experienced raters by intensity ratings on an identical computerized scale as described below. These two odor concentrations served as endpoints for eleven linear progressive mixture steps ranging from 100% fish odor (most unpleasant stimuli) to 100% rose odor (most pleasant stimuli) with a 50/50%v/v mixture as middle step. Based on a separate pilot study (n = 15, 11 women), five odor concentrations that perceptually corresponded to a linear increase in valence from the two endpoints were selected for the main experiment (see Figure 1A). This stimulus set allowed us to explore valence-dependent effects using odor stimuli positioned at the mid-range of the odor valence rating scale, a more ecological valid range than using odor stimulus positioned at the two extreme end points.

All odors were presented using a computer controlled and fully automatic olfactometer operated by the software E-Prime Professional 2.0 (Psychology Software Tools Inc., Pittsburgh, PA). The olfactometer design has been described in detail elsewhere [24]. In short, a valve control unit regulates the state of the olfactometer's solenoid valves, each of which directs a continuous airstream of 3.0 liters per minute (lpm) into an odor glass reservoir containing 10 ml of the odor in question when triggered by the valve control unit. The odorized headspace is transported to a birhinal nosepiece where one channel serves as a conduit for the odorless control flow. This control flow is directed to the nosepiece in-between odor presentations. In the nosepiece, the flow from the activated channel (odor or control) mixes with a continuous, low-flow airstream (0.5 lpm), adding up to a total airflow of 1.75 lpm per nostril. This continuous airstream masks the tactile cues that might otherwise alert the subject to channel-switching [25], [26].

### Visual Stimuli and Delivery

A face-only image (see Figure 1B, bottom left) was acquired from 55 women in the ages 35–50 using a high-resolution Canon EOS-1Ds camera. The images were taken using front-facing poses, with the subjects' hair pulled back from the face, eyes open, and assuming a neutral facial expression. In a second pilot study (n = 16, 7 female), these 55 images were rated for perceived attractiveness and age, as well as absence of emotional expression. Pilot subjects were further given an opportunity to flag images which they did not consider representative of the general population, and images with a high concordance across pilot subjects on this item were not included in the main task. This procedure was adopted to exclude faces that would draw attention towards specific unusual facial features in individual faces which would disproportionally affect the results. The eight final images were perceived to have a neutral emotional expression, were difficult to define in age, and their attractiveness was rated within two standard deviations of the mean in either direction. Individuals in the included images had a mean age of 42.25, and a perceived age of 40.06 (not significantly different from real age).

Each selected image underwent image manipulation to either increase (henceforth called ‘increased feature conditions’) or decrease (henceforth called ‘decreased feature conditions’) the appearance of wrinkles and blemishes (see Figure 1B, center). Importantly, by manipulating the faces along this single dimension, we introduced perceptual differences along both of our dependent measures, attractiveness and age perception. A total of four transformations were used, with two increasing the appearance of wrinkles and blemishes, one at a low (−25) and one at a moderate (−50) level, and two decreasing the appearance of these attributes at low (25) and moderate (50) levels. All images were presented on a 19″ TFT screen with an eye-to-screen distance of 100 cm with a visual angle of 9.77° in width and 7.04° in height. Images were presented using the stimulus presentation program E-Prime Professional 2.0 (Psychology Software Tools Inc., Pittsburgh, PA), which also controlled the olfactometer and collected subject responses.

### Procedures

Participants were first presented with an unprocessed image of a face (the reference image) for a total of 1000 ms (see Figure 1C). At the offset of the target face, odor presentation was initiated and a blank screen was displayed with a random interval between 400 to 600 ms, after which a second image of the same face with increased or decreased features (the altered image) were presented during a maximum of 2000 ms. Participants were asked to make a simple speeded decision whether the face in the altered image looked older or younger than the reference image presented before, using a response pad. Responses within the allotted 2000 ms removed the image, ended the odor presentation, and were followed by a 1000 ms blank image to remove image after effects. This procedure limited the allowed time for feature-based face processing, and second, provided a means to monitor task based attention and identify malingering participants.[3] Then, one of three questions appeared on the screen. In one third of the trials (pseudo-randomly assigned to reduce demand characteristics), participants had to assess the age, and in one third of the trials, the attractiveness of the individual in the altered image. In another third of trials, they rated the valence of the presented odor. Perceptual ratings were conducted by means of a digital visual analog scale with the anchors “extremely unpleasant”/“extremely pleasant” in the case of odor valence evaluation and “extremely unattractive”/“extremely attractive” in case of face attractiveness evaluation. The scales looked identical in each of the three questions, consisting of a visually continuous blue bar which for each question was subsequently divided into 100 subunits. In the case of the age ratings, the scale was visually anchored by the endpoints “<25” and “>60”, and ticks in steps of 5 years were placed underneath the scale to provide additional orientation and increase ecological validity. Within each question, conditions were matched in the age of the individual actors with no statistical differences between them (all *t*s<.99, all *p*s>.33). The task was repeated eight times for every possible odor (6) and facial transformation category (4) combination rendering a total of 192 trials per participant. To limit odor adaptation and habituation, an average of 12 s (+/−100 ms jitter) inter-trial-interval (ISI) was used and testing was divided into four blocks of equal duration with two minutes rest in-between each testing block.

### Data reduction and statistical analyses

To accommodate differences in variance between the age and attractiveness rating tasks, rating scale results were z-transformed before they were submitted to combined analyses. Categorical effects of modulated facial features and odor valence on attractiveness and age perception, were first analyzed by a factorial approach: for this, we recoded the two most pleasant odors into a “more pleasant” category and the two most unpleasant odors as “more unpleasant” category, excluding the middle (neutral) odor. The resulting odor categories differed significantly from each other in perceived valence (t(17) = 8.04, p<.001), and also differed significantly from the expected neutral valence point of 50 (more pleasant category: mean  = 57.95, t(17) = 4.85, p<.001, more unpleasant category: mean  = 37.90, t(17) = −7.96, p<.001). Similarly, we recoded the modulated faces as either “increased features” (−50, −25 degree of modulation) or “decreased features” (50, 25 degree of modulation) and entered these four variables into a 2×2×2 mixed-effect ANOVA with odor valence, feature strength (amount of wrinkles & blemishes), and task type (attractiveness vs. age) as fixed factors and subject variance as a random factor. Significant interactions were dissected by test-wise ANOVAs and Bonferroni-corrected Tukey post-hoc tests. To determine whether the observed modulations could be explained by linear representations of odor and face manipulations, we coded our visual and olfactory manipulations as linear regressors and conducted linear mixed effect multiple regression analyses which assessed effects of the modulation within each dimension. To control for repeated statistical testing performed on the same dataset, significant effects are reported at a significance level of *p* = .01.

Reaction time and response data often demonstrate non-spherical distributions, violating the assumptions underlying parametric inference statistics. Therefore, to allow the use of parametric testing, we log-transformed all reaction time data and arcsine-transformed participants' performance scores on the 2AFC age-discrimination task; these steps assured a normalized distribution of our data. Hereby, responses were counted as incorrect if the subjects rated a stronger morph as younger or a weaker morph as older. Nonresponses were excluded from analysis. Further, to account for the interrelatedness of accuracy and reaction time measures manifested in a speed-accuracy tradeoff, we combined these measures into a joint efficiency measure [27], [28], [29] consisting of the quotient of the transformed accuracy by the transformed reaction time measure. We hypothesized that the degree of difference between the reference image and the altered image would have an effect on task difficulty which would be reflected in higher efficiency rates with increasing differences between the two. We tested this hypothesis in a 2×2 mixed effects ANCOVA, with direction of morphing (older vs younger) and strength of morphing (strong vs weak) as the within-subject factors. Odor valence was included in the model as a linear covariate. All analyses were conducted in the R statistical computing environment (www.R-project.org), using the nlme package for linear mixed effects models, and the multcomp package for post-hoc tests.

## Results

### Analyses of Attractiveness and Age Rating Scales

Differential effects of the odor and face manipulations on age and attractiveness were observed using the factorial approach (odor by face by test interaction, F(1,119) = 10.57, p = .002), and persisted in the linear regression analysis (odor by face by test interaction, (t(695) = 2.80, p = .005). To decompose these interactions, separate models were conducted on each rating scale.

### Facial Attractiveness Ratings

**Figure 2:**
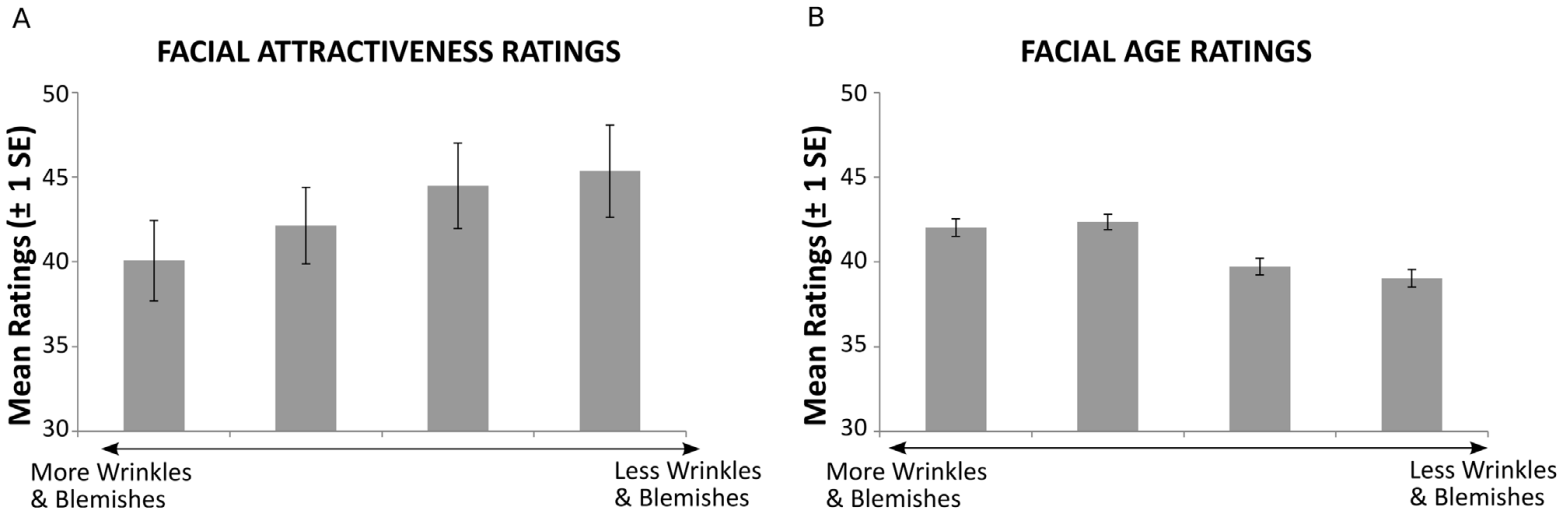
Effects of visual manipulations on attractiveness (A) and age ratings (B) averaged across odorants. Ratings were provided on a visual analog scale consisting of 100 sub segments, which in the case of age was anchored at 25 and 60 for ecological validity. Error Bars indicate +−1 SE. Across all odor conditions, a linear modulation of attractiveness and age (p<.001) were observed.

**Figure 3:**
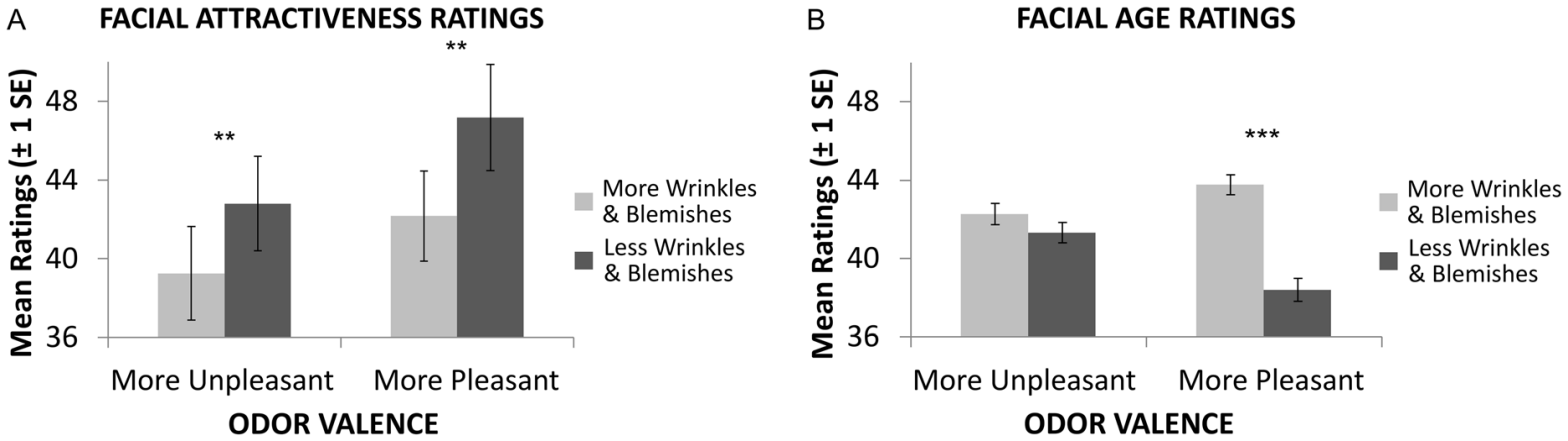
Results of factorial analyses for categorical effects of odors and facial morphing on attractiveness (A) and age (B) and ratings. Ratings were provided on a visual analog scale consisting of 100 sub segments, which in the case of age was anchored at 25 and 60 for ecological validity. Error Bars indicate +−1 SE, asterisks indicate significant differences as revealed by post hoc t-tests (*  = *p*<.05, **  = *p*<.01, ***  = *p*<.001).

Regression over all odor conditions showed a linear relationship between wrinkles and blemishes and attractiveness, (R = .21, p<.001, see figure 2A). This relationship was reflected in the factorial model [main effect of face, *F*(1,51) = 23.85, *p*<.001]. As predicted, more pleasant odors [main effect of odor, *F*(1,51) = 17.30, *p*<.001] also resulted in higher attractiveness ratings for the altered faces. No interaction between facial morphing and odor valence was observed (Figure 3A). Investigating the linear modulation across the full stimulus range, additive effects of both experimental modulations were observed: a linear increase of perceived facial attractiveness was predicted both by a linear increase of odor valence, *t*(339) = 5.19, *p*<.001, and a linear decrease of facial feature strength (amount of wrinkles & blemishes), *t*(339) = 7.05, *p*<.001.

### Facial Age Ratings

Feature strength (amount of wrinkles & blemishes) was also found to modulate age ratings, with stronger feature expression being linked to older age perception (R = −.54, p<.001, see figure 2B). While the factorial analysis reflect this effect (main effect of face [*F*(1,51) = 80.05, *p*<.001]), the effect of odors on age perception was not found to be statistically significant [*F*(1,51) = 4.01, *p* ns], but varied depending on the direction of facial feature modulation [face*odor interaction, *F*(1,51) = 39.01, *p*<.001, see figure 3B].

Post-hoc Tukey's tests demonstrated that this interaction was driven by increased and decreased feature morphs being rated significantly differently during more pleasant odor stimulation (p<.001), but not during more unpleasant odor stimulation.

The mixed regression model supported a linear effect of facial morphing strength, [*t*(339) = −7.48, *p*<.001], but not odor, [*t*(339) = −.1.06, *p* ns], on age ratings. Again, the interaction between face and odor was significant [*t*(339) = —3.23, *p* = .001].

### Odor valence ratings

As expected, the presented odor stimulus type explained variability in the odor valence ratings [main effect of odor, *F*(1,543) = 446.84, *p*<.001]. Strength of facial morphing, however, had no significant effect on odor ratings [*F*(1,543) = 6.42, *p* ns], and no significant interaction between the two variables was observed [*F*(1,543)<.001, *p* ns]. Similarly, the mixed regression model demonstrated a linear positive increase in valence ratings with increasing valence of odor mixtures, *t*(339) = 22.47, *p*<.001. No other significant effects were observed.

### Task Performance

Performance efficiency in the 2AFC task did not differ by direction of facial morphing, and no effect of odorant could be observed. Performance efficiency was, however, significantly greater when faces were more strongly morphed [*F*(1,335) = 41.55, p<.001], thus demonstrating a successful increase in perceived difference between the reference face and the altered images in synchrony with the degree of facial morphing.

## Discussion

By applying linear manipulations to odors and invariant facial features, the present study demonstrates that olfactory and visual cues can alter memory-based representations of facial attractiveness in an additive manner. While factorial analyses indicated that concurrent presentation of odors also influenced the representation of facial age, a closer examination of olfactory-visual interactions in a regression model revealed that only the integration pattern observed during attractiveness perception was consistent with the notion of olfactory-visual representation in a joint stimulus space. Given the difference between age and attractiveness judgments in their susceptibility to modulation by odors, we propose that the perceptual mechanisms which underlie olfactory-visual integration of invariant facial features are specific to affective processing.

Our findings replicate and extend reports from previous studies, which suggest that emotionally-valenced odors have the ability to influence the perceived attractiveness of presented faces [12], [13] even when the subject is instructed to focus on the face alone. While previous studies have looked at extreme cases of odor valence and investigated effects on one outcome variable, we can demonstrate here that increases in observed odor valence are specific to attractiveness perception and translate directly onto ratings in an additive fashion: as expected, modulations of both visual properties and odor valence were associated with linear effects on attractiveness perception.

While multisensory integration at the early perceptual level is usually associated with superadditive effects [30], [31], affective responses to multisensory input are thought to be independent from the availability of attentional resources [32] and thus characterized by linear summation. Given the strong affective component of attractiveness ratings, which tend to cluster along a valence dimension subserving approach and avoidance behavior [33], the observed linear integration pattern thus represents a typical response to affective evaluation of multisensory input. This combination into a joint stimulus space is thought to result from supramodal affective back-projections from prefrontal cortex, which have been shown to modulate emotional object evaluations [34]. Equally, the strong anatomical overlap between affective prefrontal areas and secondary olfactory cortex [35], provides a unique link between olfactory object and valence perception [36], [37], [38]. Taken together, our findings provide strong support for affectively-mediated olfactory influences on face perception, which are unique to evaluation tasks possessing a shared emotional component.

By contrast, the effect of odors on age perception demonstrated an interaction on the factorial level. While face morphs of opposite direction were rated as similar in age under more unpleasant odor conditions, they were clearly rated as different in age under more pleasant odor conditions. Linear regression analysis confirmed the interaction pattern and linear judgment of facial aging cues, and did not support a linear combination of odors and faces. Age perception has been reported to represent a higher cognitive load relative to emotion-based evaluations [18], and various studies have to date reported an interference of emotionally emotionally-valenced stimulus material with effortful cognitive processing [39], [40], [41]. Consistent with evidence suggesting that age-perception is a learned higher-order cognitive mechanism [14], [15], we therefore propose that in the context of age-perception, unpleasant odors may constitute a threat signal which elicits automatic allocation of attention to affective cues [42], so emotionally-irrelevant aging cues likely lose salience by comparison. During pleasant odor stimulation, however, more attentional resources might be allocated to the face perception task, resulting in an increased difference in perceived age between increased and decreased feature conditions. These findings are consistent with the idea that odor and attractiveness perception, but not odor and age perception, feed into a common affective neural system and therefore jointly affect behavioral responses. Future studies will need to focus on manipulating the attentional load and distraction components in order to more comprehensively describe the demonstrated interaction pattern, and specifically test hypotheses relating to the neural mechanisms underlying the observed effects.

Our study design resulted in a number of inherent drawbacks which should be taken into consideration. Most importantly, it is not possible in the context of the present experiment to quantify the extent and direction of a potential bias introduced by our choice of speeded response task during presentation of the morphed image. A two-step response format was adopted to effectively limit analytical exploration times of the faces to a time that would not allow for extended detailed inspection of the visual images. This resulted in a higher difficulty level for the memory-based rating task, which is a prerequisite for the observation of multisensory integration effects. However, given the conceptual similarity between the older/younger decision in the speeded task and the subsequent rating, we cannot rule out that our results may have been affected by conceptual priming effects on age, but not attractiveness, decisions during encoding. Given that the homogeneity of the facial battery used in this task restricted us to this task choice, future studies should use a more diverse battery to open up possibilities for modifications of the experimental design, and a detailed investigation of the influence of the choice of speeded response task on the outcome of these ratings. Doing so would further allow for investigations of sex-dependent differences in the study sample or stimulus material, as have been suggested by previous studies [43]. To optimize our experimental power towards the detection of linear effects, we opted to prioritize a higher number of repetitions for crossmodal conditions and not include a condition that showed the unmorphed picture as the target image (ie. a visual stimulus repetition), and a no odor condition without concurrent face presentation. As a result, we do not have unimodal reference ratings of odors or face stimuli from our study sample. We do not, however, have any reason to suspect a systematic variation between the study and pilot samples. Moreover, our study was restricted to a stimulus set of female faces.

Finally, due to increasing evidence for specific odor associations across the life span [44], [45], [46], we chose to restrict the age range of the face battery to middle-aged individuals. Future studies should investigate whether these effects may be modulated by the age range of both the facial stimulus battery and the observers.

In conclusion, our study demonstrates that linear olfactory enhancement of facial perceptual judgments occurs for attractiveness, but not age ratings. These integration mechanisms are unlikely to reflect basic bottom-up sensory processes, but rather, valence-dependent supramodal associations, analogous to the perceptual principles regulating audio-visual emotional integration [47], [48]. Olfactory influences on analytical cognitive processing of faces, as observed in age judgments, follow a pattern that is indicative of cognitive interference. While olfactory effects on person perception have long been neglected in the laboratory, this study stresses that such effects likely have an important effect on the affective connotation of real-life social interactions and deserve further attention.
